# Huir del nazismo: Alejandro Lipschütz y los casos de Alfons Nehring y Käte Pariser

**DOI:** 10.1590/S0104-59702023000100059

**Published:** 2023-10-23

**Authors:** Marcelo Sánchez Delgado

**Affiliations:** i Centro de Estudios Culturales Latinoamericanos Facultad de Filosofía y Humanidades Universidad de Chile Santiago de Chile Chile historia.mjsd@gmail.com Profesor asistente, Centro de Estudios Culturales Latinoamericanos/Facultad de Filosofía y Humanidades/Universidad de Chile.Santiago de Chile – Chile historia.mjsd@gmail.com

**Keywords:** Nazismo, Alejandro Lipschütz (1883-1980, Alfons Nehring (1890-1967, Käte Pariser (1893-1953, América Latina, Nazism, Alejandro Lipschütz (1883-1980, Alfons Nehring (1890-1967, Käte Pariser (1893-1953, Latin America

## Abstract

Este trabajo estudia una red de acogida para científicos judíos desplazados por el nazismo a partir del archivo de Alejandro Lipschütz, fisiólogo que vivió en Chile desde 1926. A partir del contexto de la persecución antisemita y la forma en que afectó a la ciencia y a la universidad alemanas se analizan las cartas remitidas hacia y desde Lipschütz entre 1935 y 1936, con especial atención a personas que lo contactaron para huir de Alemania y que veían en América Latina una posibilidad. Proponemos que se trata de una red de agencias personales, cargada de subjetividades e intimidad, que debía tener en cuenta el antisemitismo y la xenofobia académica local.

## Abstract (en)

This paper studies a shelter network for Jewish scientists displaced by nazism from the archive of Alexander Lipschütz, a physiologist who lived in Chile since 1926. From the context of the anti-Semitic persecution and the way in which it affected German science and their universities, we have analyzed letters sent to and from Lipschütz between 1935 and 1936, with special attention to people who contacted him to flee Germany and considered Latin America as a possibility to live. We suggest this was a network of personal agencies, charged with subjectivities and intimacy, which had to take into account local anti-Semitism and academic xenophobia.

## La distopía racial alemana y el exilio científico

La idea de una unidad racial capaz de abolir los conflictos de clase tuvo un rol fundamental en la ideología nazi. Aunque la “comunidad del pueblo” (*Volksgemeinschaft*) fue una imagen impulsada para consagrar la unidad como alianza interclasista para lograr el triunfo en la Primera Guerra Mundial, en el seno del nazismo se transformó en un concepto clave del imaginario político que dio fuerza al antisemitismo (Morente, 2006). El judío fue transformado en una fuerza venenosa y maligna, a la que se culpaba de la inflación, la crisis económica, la inercia de la burocracia estatal, el capitalismo financiero voraz, la degeneración de la raza, la decadencia cultural y la destrucción de los valores tradicionales (Cruz, 1989), males que pusieron a los judíos en el rol de un doble metafísico de la comunidad del pueblo ario (Burrin, 2007).

Tras asumir el poder en 1933, Hitler ordenó un proceso de reorganización de la vida pública, la llamada “homogeneización” (*Gleichschaltung*). Una de las leyes de este proceso fue la “Ley para el restablecimiento del servicio civil profesional”, del 7 de abril de 1933, que ordenaba la exclusión de los no arios y los opositores políticos en todos los puestos del Estado. Este impulso al antisemitismo fue fundamental para un proceso de purgas y reconstrucción ideológica que afectó a la universidad y a la ciencia alemanas. Es un fenómeno bien estudiado en sus efectos, entre los que se suele destacar el impulso decisivo que estas expulsiones y posterior exilio de científicos dio a la física y al desarrollo de la energía nuclear en el espacio geopolítico de las fuerzas aliadas en guerra contra el eje nazi-fascista (Ball, 2014).

Este trabajo se propone analizar una red latinoamericana de acogida para judíos alemanes ligados a la ciencia y a la academia. Nuestro punto de entrada para conocer esta red es Alejandro Lipschütz (1883-1980), médico letón radicado en Chile desde 1926 que fue una figura de importancia en el indigenismo latinoamericano y en la antropología chilena, entre otras facetas (Parraguez, 2017). Era muy activo en una red epistolar que incluía a Sigmund Freud, Ivan Petrovich Pavlov, Gregorio Marañón, Albert Schweitzer, Juan Comas, Manuel Gamio, entre otros. Marxista fiel al Partido Comunista chileno, tenía una clara conciencia de su identidad judía y se mantuvo al tanto de los acontecimientos en Europa, en principio como una natural preocupación por su hija que residía en España, y luego con activa preocupación por los judíos alemanes y centroeuropeos.

Por lo anterior, no resulta extraño que científicos y científicas alemanes se dirigieran a Lipschütz por vía epistolar en busca de consejos o un puesto de trabajo en Sudamérica. Se trata, entonces, de mirar hacia esta red, tomando como punto de entrada las gestiones e intereses de Lipschütz y sus interlocutores en ella. Iniciaremos con un contexto del proceso de purga y exilio que sufrió la universidad y la ciencia alemanas, luego expondremos algún apunte sobre la legislación y prácticas con que se reguló la migración judía hacia Chile en la época del nazismo. Como tema central volveremos sobre la figura de Lipschütz, para analizar el corpus de documentación inédita que ha motivado esta investigación. Así, el objetivo y tema principal de este trabajo es analizar la documentación inédita del archivo personal de Alejandro Lipschütz bajo la óptica de una red de acogida para científicos judíos y judías que huían del nazismo.

Desde hace pocos años la historiografía anglosajona ha relevado un campo complejo y sofisticado de estudios sobre el conocimiento migrante, área que comprende el proceso migrante tanto de las ideas como de las personas y su capital cultural en un nuevo contexto, y que aborda tanto el registro histórico como el tiempo presente (Westerman, Erdur, 2020). Este trabajo intenta enriquecer este campo aportando un análisis contextualizado de materiales novedosos y de una gran riqueza interna, hasta cierto punto poniendo al lector o lectora en contacto la experiencia que se desprende de la documentación, como sugiere la deriva post narrativa (Philainen, 2019). Así, nuestra tarea es humilde pero no por ello menos importante que la de un determinado campo de estudios que mira a Latinoamérica como caso en que solo la visión desde el norte puede otorgar sentido. Este trabajo afirma la posibilidad de mirar el mundo desde el sur, integrando a nuestra historiografía los problemas, temas, y conflictos en calidad de otro centro, entre muchos otros, y no de caso que solo se sustentaría en la mirada de una historiografía que se piensa y se escribe en otras latitudes y lenguas.

## Persecución antisemita y exilio científico: refugiados indeseables

La comunidad académica y científica alemana recibió mayoritariamente con entusiasmo la depuración racial promovida por el nazismo, ya que estaba muy predispuesta a “aceptar, en diversos grados, el espíritu alemán en la academia, un concepto que abarcaba el nacionalismo agresivo, el racismo, el antisemitismo y el rechazo de la objetividad en la investigación y la docencia” (Remy, 2016, p.52). El mismo Remy (p.57) aporta que “a finales de 1938, Alemania (incluyendo Austria) había perdido el 39 por 100 de sus profesores universitarios”. Utte Deichman ha llegado a desglosar las pérdidas de docentes e investigadores por áreas de la ciencia, señalando:

Con un 25% de despidos y emigraciones entre los profesores e investigadores universitarios en los Institutos Kaiser Wilhelm, la química se vio fuertemente afectada; entre las subdisciplinas de la química, la química física sufrió las pérdidas más considerables (36%). Tanto la bioquímica como las matemáticas sufrieron pérdidas de alrededor del 33%, mientras que la biología (zoología, botánica y genética no humana) se vio menos afectada (8%). El impacto en la física estaba en algún punto entre esos extremos, pero no tenemos cifras concretas. Después de las matemáticas, la bioquímica alemana fue la que más investigadores importantes perdió, entre ellos varios ganadores presentes y futuros del Premio Nobel (Deichman, 2019, p.44).

Las leyes de Nurenberg de 1935 llevaron a una depuración racial casi total de la universidad y la ciencia alemanas, al expulsar a personas casadas con judíos o judías. Consecuentemente, el periodo de 1936 a 1939 fue un punto de inflexión ya que el régimen aceleró la persecución de los judíos y se preparó para la guerra (Deichman, 2019). Así, para los científicos judíos, desde 1933 en adelante la necesidad de salir de Alemania se fue intensificando, hasta convertirse en un propósito de supervivencia.

El proceso de exilio afectó a personas de un estatuto muy diferente. Están los casos de científicos y académicos que ya tenían un renombre internacional y eran celebridades en su campo disciplinar como Albert Einstein, Max Born, Martin Buber, Theodor Adorno, Max Horkheimer, Karl Mannheim, Ernst Cassirer, entre muchos otros (Morente, 2006, p.70), pero el proceso afectó por igual a los escalones más bajos del profesorado universitario, estudiantes de diferentes niveles, investigadores en etapas iniciales de formación y técnicos. El destino del exilio también resultó problemático y como se ha señalado “con el triunfo del franquismo y el inicio de la Segunda Guerra Mundial, prácticamente no quedó territorio continental en Europa donde refugiarse” (Cebolla i Moll, 2002, p.53). En todo caso, desde 1933 en adelante, las personas expulsadas de la universidad y la ciencia alemana comenzaron a buscar rumbo en el ancho mundo, buscando oportunidades de trabajo en lugares tan diferentes como Turquía, Brasil, EEUU y la Unión Soviética, entre otros.

En Chile, durante la década de 1930, la política de migración para personas judías osciló entre el rechazo, la acogida, prohibiciones explícitas o veladas, períodos de acogida, y finalmente un cierre total de fronteras. La depresión de 1929 instaló políticas restrictivas y sobre ellas comenzaron a instalarse las presiones pro y antisemitas (Hernández Ferrada, 2015). Gracias a dos buenos trabajos de Brahm García y Montes Arrazota (2012, 2013) sabemos con precisión sobre acuerdos restrictivos por parte del gobierno del presidente Alessandri (1932-1938) y la política mucho más flexible que inició el gobierno del Frente Popular en 1938, aunque debido a acusaciones de corrupción la apertura terminó con una medida radical como la suspensión indefinida del visado de pasaportes, con la explícita mención a los cónsules de “tomar las medidas discrecionales para impedir el embarque de judíos” (Brahm García, Montes Arrazota, 2012, p.914).

## Alejandro Lipschütz: la época de ascenso del nazismo y las redes de apoyo a científicos judíos

En la última década ha surgido un conjunto de estudios monográficos sobre Lipschütz, que abordan la multifacética obra con que destacó internacionalmente en ámbitos como endocrinología, oncología, fisiología, antropología, etnografía, vida universitaria, difusión de la ciencia e indigenismo (Yuing, Carvajal, 2012; Ruperthuz, 2014; Vergara, Gundermann, 2016; Samaniego, Rodríguez, 2017; Vetö, 2017; Vetö, Sánchez, 2017; Parraguez, 2017; Chiappe, 2018; Sánchez Delgado, 2019).

Alexander Lipschütz Friedman nació en Riga, capital de la actual Letonia, el 28 de agosto de 1883, ciudad que era parte del Imperio Ruso. Provenía de una familia de origen judío alemán y comenzó sus estudios de medicina en 1902, pero se interrumpieron ya que se involucró en la revolución rusa de 1905 y finalmente terminó su doctorado en Göttingen en 1907. Fue discípulo de Heinrich Waldeyer (1836-1921), un pionero de la biología celular y de Oscar Hertwig (1849-1922), discípulo sobresaliente de Ernst Haeckel. Consiguió una posición de ayudante en Gottingen desde la que colaboró con el doctor Max Verworn (1863-1921), otro destacado discípulo de Haeckel, y con August Pütter (1879-1929), fisiólogo y zoólogo alemán. En 1914 publicó *Warum wir Sterben* (Porque morimos), una obra que le dio fama internacional y que despertó el interés de Thomas Mann (Manríquez, 1993) y Sigmund Freud (Vetö, Sánchez, 2017). En una breve estadía en Berna durante la Primera Guerra Mundial, fue ayudante de Eugen Steinach (1861-1944), un pionero de la endocrinología, y de allí en adelante sus investigaciones biomédicas se relacionaron principalmente con esta disciplina. En 1919 fue contratado por la Facultad de Medicina de la Universidad de Dorpat, Estonia, la que dejó en 1926 para trasladarse a la Facultad de Medicina de la Universidad de Concepción en el sur de Chile (Lipschütz, 1970).

La relación de Lipschütz con la Universidad de Concepción se deterioró debido a diferencias económicas y derivó en un litigio legal entre 1935 y 1937. En este periodo evaluó seriamente la posibilidad de trasladarse a España, pero los sucesos europeos lo llevaron a establecer una alianza con un médico y político liberal chileno, el doctor Eduardo Cruz Coke (Cárcamo Gebhardt, 2021), quien logró que se estableciera un Instituto de Medicina Experimental a cargo del Estado, que fue puesto bajo la dirección de Lipschütz (Dosne Pasqualini, 2014). Así, desde 1926 hasta 1937 Lipschütz estuvo a cargo del Instituto de Fisiología de la Universidad de Concepción y desde 1938 a cargo del Instituto de Medicina Experimental en la capital del país.

En el contexto del apoyo a los científicos judíos contamos con una carta de Lipschütz, fechada el 22 de mayo de 1936, dirigida al Academic Assistance Council (AAC en adelante). El AAC surgió en mayo de 1933, apenas comenzó la exclusión de los judíos del Estado alemán y convocó a notables figuras de la escena intelectual británica y europea. Sus impulsores fueron el economista William Beveridge (1879-1963), el físico judío húngaro Leó Szilárd (1898-1964) y su primer director, el físico neozelandés Ernst Rutherford (1871-1937), conocido como Lord Rutherford. En esta misiva, Lipschütz dio cuenta a la AAC de sus gestiones para conectar con esta institución a Mariano Picón Salas, un escritor y político venezolano que había residido en Chile entre 1924 y 1936 y que ocupaba en ese momento el cargo de Superintendente de Educación de Venezuela. En la carta, Lipschütz transcribió un fragmento de la comunicación de Picón Salas, quien señalaba: “Estoy muy interesado en tu proposición de ayudar a los profesores no arios que han sido violentamente retirados de sus cátedras en Alemania. Si pudiéramos tener una lista de estos profesores, algunos de ellos podrías ser elegidos para las diferentes posiciones de enseñanza que serán creadas en Venezuela” (Lipschütz, 22 mayo 1936). Como puede constatarse de entrada, Lipschütz estaba preocupado por la situación de los judíos alemanes expulsados de las instituciones científicas y de la universidad y dispuesto a articular redes con colegas y conocidos que estaba en situaciones de poder en el amplio espacio geográfico de sus redes, que abarcaba personas con puesto provistos de algún poder en distintas latitudes.

El 23 de junio de 1936 está fechada la respuesta de la AAC, firmada por el secretario de esta institución, Walter Adams, un destacado historiador de la London School of Economics en ese periodo. Adams comentó a Lipschütz: “Muchas gracias por su carta del 22 de mayo contándome el interés mostrado por el profesor Mariano Picón-Salas en un trabajo para los académicos alemanes desplazados. Esperamos sinceramente que algo pueda surgir como resultado de su generosa acción al interesarlo” (Adams, 23 jun. 1936).

La carta también da cuenta del paso de esta fundación, dedicada a apoyar a los profesores y científicos alemanes, a otra con una dimensión mayor, hecho que ocurrió a mediados de 1936 y que Adams comentó:

Es evidente que existe una necesidad continua de los servicios de organismos como el Consejo de Asistencia Académica y, por esta razón, Lord Rutherford recientemente tomó la decisión de transformar el Consejo en una organización más permanente, la Sociedad para la Protección de la Ciencia y el Aprendizaje. Puede interesarle ver la declaración adjunta emitida por Lord Rutherford dando una explicación de estos planes (Adams, 23 jun. 1936).

Efectivamente, otro documento del archivo personal de Lipschütz es la mencionada declaración, un folleto impreso de cuatro páginas de “The Society for the Protection of Science and Learning” (Zimmerman, 2006). El folleto señala que la AAC ya había colocado, en trabajos estables, a 363 de los primeros setecientos profesores alemanes desplazados. Estas acciones, que podrían haber sido parte de un auxilio circunstancial, no podían terminar. Según el folleto: “La devastación de las universidades alemanas aún continúa; no solo los profesores universitarios de ascendencia judía, sino muchos otros que se consideran ‘políticamente poco confiables’ están siendo impedidos de hacer su contribución a la causa común de la erudición” (AAC, mar. 1936).^1^

La carta de Adams informaba también del traslado a Londres, a las oficinas contiguas a las de la AAC, de la Asociación de Emergencia de Científicos Alemanes en el Extranjero (Notgemeinschaft Deutscher Wissenschaftler im Ausland) (Löhr, 2014). Adams (23 jun. 1936) señaló que dicha sociedad “ha hecho la mayor parte del trabajo en la colocación de académicos alemanes en los países de América del Sur”. Efectivamente, Lipschütz recibió también una circular de la Asociación de Emergencia de Científicos Alemanes en el Extranjero que informaba de la instalación de académicos alemanes en el extranjero: 12 en Colombia, nueve en Panamá, ocho en Turquía, cuatro en Rusia, tres en Italia, tres en Irán, dos en Suiza, dos en Brasil, un en Albania, un en Perú, un en Chile, un en Argentina y un en Lituania (Notgemeinschaft…, 1935-1936).

Como era corriente en el ambiente científico de gran parte del siglo XX, Lipschütz solía intercambiar separatas de artículos científicos con investigadores de Europa y EEUU. En una comunicación de este tipo, fechada el 27 de agosto de 1936, del Profesor J. Freud (27 ago. 1936) del Laboratorio Fármaco Terapéutico de Amsterdam le comentó: “Te extrañé en el congreso en Rusia el año pasado. Todavía lo estamos haciendo relativamente bien aquí. ¿Existe tal vez una oportunidad para alojar refugiados en Chile? El Prof. Laqueur realmente se esforzó mucho por ellos, pero siguen llegando nuevos candidatos. Con los más cordiales saludos tu devoto. J. Freud”.

Como leemos, la carta pregunta directamente por la posibilidad de recibir refugiados en Chile.^2^ Lipschütz (16 oct. 1936) respondió con un panorama poco alentador y transmite su propio fracaso: “En su carta pregunta si existe la posibilidad de alojar refugiados aquí. He enfrentado esta pregunta muchas veces para poder acomodar refugiados alemanes aquí en Chile y en otras repúblicas sudamericanas. Desafortunadamente no tuve éxito, con la excepción de un solo caso”.

Lipschütz agregó también que los médicos locales solían denunciar un exceso de profesionales compitiendo por una clientela reducida:

Actualmente existe una decidida antipatía en Hispanoamérica a la emigración de colegas extranjeros, debido en parte a la intensificación de la lucha por la existencia en la profesión médica. Incluso, algunos de los tratados que regulaban el reconocimiento mutuo del ejercicio de la medicina entre varias repúblicas sudamericanas han sido rescindidos. Todo es muy triste y no se sabe cómo resultarán las cosas (Lipschütz, 16 oct. 1936).

A lo anterior debían sumarse rangos variables de antisemitismo en la sociedad chilena, elemento que Lipschütz señalará explícitamente a sus interlocutores a propósito de dos casos en que es interpelado personalmente por científicos alemanes en busca de ayuda y consejo para encontrar un trabajo en Chile o en algún otro país sudamericano.

## Alfons Nehring busca una cátedra: “Fui jubilado por la nueva ley porque soy un supuesto no ario”

La precaria situación de los académicos alemanes expulsados de sus cátedras resulta bien retratada por la misiva que el filólogo Alfons Nehring envió a Lipschütz, fechada el 5 febrero de 1936. Se trata de una carta en la que se deja ver una situación desesperada. En primer lugar, Nehring solicita comprensión: “Perdóneme si, como un completo extraño para usted, me permito recurrir a usted después de que me dieron su nombre desde América” (Nehring, 5 feb. 1936), le advierte, y pasa a exponer su situación:

Fui profesor ordinario hasta 1933. Era profesor de lingüística comparada en la universidad local y luego fui jubilado por la nueva ley porque soy un supuesto ‘no ario’. En mi caso, eso significa que mi madre proviene de una familia judía. Dado que en 1933 solo tenía 43 años, mi pensión es, por supuesto, muy pequeña. Desde entonces he trabajado con suma diligencia en mi quehacer científico. Desafortunadamente, sin embargo, las oportunidades de publicación ahora son cada vez menores. Algunas de las revistas no aceptan contribuciones de personas que no sean arias, y recientemente tuve una experiencia muy desagradable con una contribución solicitada con urgencia para un artículo conmemorativo, en el que trabajé durante un año y que cuando estaba terminado, según las leyes de Nuremberg, ya no se pudo aceptar. Eso hace imposible la existencia para un científico, y además, a los 45 años y con una pequeña pensión, no te puedes quedar de brazos cruzados. Así que ahora tengo que intentar todo lo que pueda para encontrar un nuevo trabajo en el extranjero, y por eso me recomendaron que investigara sobre posibles oportunidades en Chile. Así que me gustaría pedirle educadamente si fuese tan amable de hacerme este favor como un colega (Nehring, 5 feb. 1936; énfasis en el original).

Nehring complementó esta presentación con una descripción de sus áreas de trabajo presentándose como un especialista en lingüística comparada, filología, filosofía del lenguaje, arqueología y etnología, que buscaba una posición académica en Chile u otros países de la región. Nehring solicitó a Lipschütz consejo y orientación: “Le agradecería mucho si fuera tan amable de decirme si hay perspectivas y, en caso afirmativo, cuáles, y si también pudiera darme algún consejo si fuera necesario. Seguro que usted tiene amplias relaciones. Por cierto, también sería valioso para mí si pudiera mostrarme o brindarme una posibilidad en otros países” (Nehring, 5 feb. 1936) y culminó su carta, apelando otra vez a su situación personal en Alemania:

Disculpe las molestias. Sin duda comprenderá y sentirá que, en las circunstancias imperantes, uno debe hacer todo lo posible para encontrar nuevamente una posición académica. Ya es bastante triste que te veas obligado a hacerlo después de haber conseguido una posición a través de tu trabajo y después de haber sido un buen alemán toda tu vida. Estoy seguro que empatizarás conmigo (Nehring, 5 feb. 1936).

Alfons Nehring nació en Silesia en 1890 y realizó sus estudios universitarios en Breslau y en Berlín, recibió el doctorado en 1915 y la habilitación en 1923. Desde 1930 era el titular de la cátedra de lingüística comparada en la Universidad de Würzburg, hasta que fue jubilado prematuramente en 1933. Aunque Nehring era un ferviente católico dispuesto a enrolarse en la “Asociación del Reich de ciudadanos cristianos alemanes de ascendencia no aria o no aria pura” y reconocer al régimen (Maas, 4 mayo 2018), la carta que dirigió a Lipschütz en 1936 muestra que su situación comenzaba a ser desesperada, ya que no tenía trabajo ni podía seguir publicando. El caso de Nehring devela los excesos del racismo arianizante al expulsar de la comunidad científica alemana a un católico cuya falta era tener madre judía, y que además se consideraba a sí mismo y para todo efecto, “un buen alemán”.

La carta fue respondida por Lipschütz el 19 de marzo de 1936, con una combinación de franca acogida y un diagnóstico pesimista para las aspiraciones del filólogo alemán. Lipschütz (19 mar. 1936) le advirtió que:

En las universidades sudamericanas difícilmente se puede hablar de investigación filológica y de enseñanza científica de la filología, salvo casos muy aislados. Por el momento, hay una falta de comprensión científica de la filología en América del Sur, y en las universidades esto se reduce a las clases de lengua y literatura. Hay más comprensión para la etnología, aunque muchas veces está en manos de autodidactas.

A pesar de este negativo panorama inicial, Lipschütz se puso inmediatamente en acción y legó a Nehring algunos consejos que consideraba adecuados. Le traspasó la dirección postal del etnólogo alemán Max Uhle (1856-1944) que al momento era profesor en Quito, Ecuador, sin dejar de señalar que “las condiciones allí actualmente son tan inestables que es muy poco probable que el señor Uhle pueda transmitir algo de su interés” (Lipschütz, 19 mar. 1936). Además, comentó a Nehring que se había tomado la libertad de remitir una copia de su carta a “un joven filólogo español muy capaz, Profesor Amado Alonso” (Lipschütz, 19 mar. 1936), que era profesor del Instituto de Filología de la Universidad de Buenos Aires. Como es conocido, Amado Alonso (1896-1952) vivió en Buenos Aires entre 1927 y 1946, y desde su llegada al país participó activamente de la vida cultural bonaerense. Sin embargo, Lipschütz sugirió a Nehring otros destinos para proseguir su vida académica. En principio, le comentó que “si no pierdes tu pensión emigrando a España o Portugal, sin duda te aconsejaría que te mudes a uno de estos países, y en especial a España” (Lipschütz, 19 mar. 1936). Luego apuntó a la Unión Soviética, y para ello ofreció facilitar el contacto con el profesor Max Vasmer (1886-1962), filólogo de la Universidad de Berlín, a quien Lipschütz conocía por haber sido colegas en la Universidad de Dorpat.

Lipschütz escribió además a Mariano Picón Salas, superintendente de educación de Venezuela, que había vivido varios años en Chile, y le presentó el caso de Nehring, señalando que:

Yo recibo frecuentemente cartas de profesores universitarios alemanes que perdieron sus cátedras por razones raciales. Me permito transmitirle la traducción de una de esas cartas, se trata del profesor titular Nehring, de lingüística comparada. Tal vez podría ser útil a ustedes en Caracas. Sería una adquisición muy considerable para la enseñanza universitaria en Venezuela (Lipschütz, 15 abr. 1936).

Al final de la misiva, Lipschütz muestra en parte las emociones e intenciones que le llevaban a esta tarea: “Le ruego tenga la bondad de disculpar que me he tomado la libertad de escribirle este asunto. Pero estoy deseoso de ayudar aquellos alemanes que tienen que sufrir tanto de las brutalidades del régimen nazista” (Lipschütz, 15 abr. 1936).

Lipschütz también escribió al profesor Arturo Piga, del Instituto Pedagógico de la Universidad de Chile, que había formado parte de una alta comisión chilena para la cooperar en la reforma educativa de Costa Rica (Mata-Rivera, 2007), solicitándole estudiar un cargo para Nehring en ese país:

Supongo que en Chile no habría ninguna posibilidad de colocación de este profesor en vista de las dificultades económicas por las cuales pasa la Universidad de Chile. Así que me vino la idea de si tal vez habría interés para la contratación de tal profesor en la nueva Universidad, en Costa Rica en cuya organización Ud. y el colega Galdames han participado en forma tan sobresaliente. Sería el doctor Nehring una adquisición muy considerable para una joven universidad (Lipschütz, 9 mar. 1936).

Antes de que Nehring respondiera la primera contestación de Lipschütz, este volvió a escribirle el 23 de abril de 1936 dando cuenta del resultado de algunas acciones emprendidas, pero fundamentalmente le comunicaba con pesar que:

Durante el último mes he estado buscando principalmente en Santiago. Desafortunadamente, en lo que respecta a la filología, se puede esperar muy poco de las condiciones aún muy incompletas allí. Hoy recibí una carta del profesor Amado Alonso en Buenos Aires. De manera conmovedora, el Prof. Alonso ha dado todos los pasos posibles, tanto en Buenos Aires como en La Plata, pero sin éxito, ya que aún no se dispone de los recursos correspondientes (Lipschütz, 23 abr. 1936).

En esta carta Lipschütz ofreció a Nehring la dirección postal de Rudolf Jaffé (1885-1975), para conseguir un consejo directo sobre la situación en Venezuela, ya que Jaffé, médico judío alemán, había sido director del Instituto de Patología y Bacteriología del Hospital Berlín Moabit hasta que lo nazis lo obligaron a abandonar esta posición en 1934 y desde 1936 estaba instalado en Caracas como director del Instituto de Patología del Hospital Vargas de esa ciudad. En resumen, Lipschütz solicitó un puesto para Nehring en Costa Rica, Buenos Aires y Venezuela, convencido que en Chile las posibilidades eran nulas, tanto por la crisis económica como por el carácter inmaduro de ciertas disciplinas en el país. Lipschütz intentaba además respaldar el subido autoconcepto del propio Nehirng presentándolo como una “notable adquisición” y “una adquisición muy considerable para la enseñanza universitaria”, lo que remite también a una sociabilidad respetuosa entre varones dedicados a la vida académica.

La única interlocución de Nehring a las gestiones de Lipschütz conservada en este archivo es una misiva del 29 de mayo de 1936. La carta transmite emociones que expresan ansiedad y pesar. El profesor alemán señaló “ha sido una carga pesada para mi alma durante mucho tiempo que aún no le haya agradecido su primera carta amable” (Nehring, 29 mayo 1936) y que “ahora he recibido su segunda carta y me avergüenzo aún más de mi largo silencio. Es realmente conmovedor cómo tú, como un completo extraño, te interesas por mí. Esta es una voluntad humana de ayudar, para la cual las palabras de agradecimiento son demasiado secas” (Nehring, 29 mayo 1936). Las emociones de Nehring parecen estar casi desbordadas en este momento.

Nehring lamentó el fracaso de las gestiones con Alonso en Buenos Aires y finalmente volvió a exponer un angustioso estado de ánimo. Consideraba que su búsqueda de nuevos horizontes vitales había entrado en una nueva etapa:

En general, ahora estoy en el proceso de abordar estas preguntas de manera más sistemática. Usted probablemente sabe que se necesita tiempo y concentración de pensamiento. Ambos han estado ausentes hasta ahora. Pero ahora tengo un poco más de aire. Y si uno sabe que es tan servicial y tan enérgicamente apoyado, abordas el asunto con aún mayor entusiasmo. Me tomaré la libertad de informarte de lo que estoy logrando gracias a tus amables recomendaciones y consejos, por lo que te agradezco nuevamente (Nehring, 29 mayo 1936).

En esta misiva se intercala también una situación tragicómica muy propia del pequeño mundo académico. Como se recordará, Lipschütz había recomendado a Nehring ponerse en contacto con Max Vasmer de Berlín para explorar contactos en la Unión Soviética. Nehring le advierte a Lipschütz que “hice una reseña de un libro de Vasmer hace muchos años y planteé algunas objeciones e inquietudes. Aunque esto se hizo de una manera estrictamente objetiva y con pleno reconocimiento del logro de V., como sé, V. tomó esta crítica muy en serio, por lo que realmente no puedo dirigirme a él” (Nehring, 29 mayo 1936).

A pesar de esta advertencia, Lipschütz escribió a Max Vasmer, director del Instituto para la Filología Eslava de la Universidad de Berlín, el 19 de junio de 1936. Lipschütz y Vasmer habían sido colegas en la Universidad de Dorpat y conservaban una mutua simpatía. En la breve misiva Lipschütz comunica directamente a Vasmer que:

Dado que estoy ansioso por ayudar al profesor Nehring y a muchos otros que se encuentran en una situación similar, le he sugerido que considere mudarse a Rusia, donde asumo que puede haber necesidad de científicos filólogos. Desafortunadamente, la filología en América del Sur aún no ha madurado lo suficiente como para crear puestos docentes como el que el Profesor Nehring podría considerar. Supongo que usted, querido Sr. Vasmer, mantiene contacto científico con los colegas rusos y me permito pensar en el Sr. Nehring a este respecto (Lipschüz, 19 jun. 1936).

Vasmer respondió la carta de Lipschütz el 31 de julio de 1936, y, junto con advertir al posible migrante de las condiciones especiales de la vida en la URSS, requirió ayuda para otro científico judío exonerado:

Basándome en mi experiencia, aconsejaría a los que van allí que mantengan una ciudadanía extranjera y no tomen la rusa. De lo contrario, corre el riesgo de ser arrestado repentinamente y expulsado allí. También me gustaría hacer algo por nuestro colega local, el Prof. Ernst Lewy. Hasta ahora le he aconsejado contra Rusia y en la conversación con él también pensé en su universidad. Pero debo concluir de su carta que las perspectivas allí son bastante malas (Vasmer, 31 jul. 1936).^3^

Como podemos apreciar, cada vez que Lipschütz intentaba acudir a sus contactos europeos, era abordado con nuevas solicitudes e insinuaciones para que lograra puestos de trabajo académico en el país o en la región para algún otro académico o científico en problemas en Alemania, lo que nos habla de la intensidad de las emociones y energías puestas en juego en estos intercambios epistolares.

Finalmente, contamos con una última misiva de Lipschütz a Nehring, fechada el 2 de septiembre de 1936, en la que sugiere a Nehring investigar posibilidades en Rusia, al mismo tiempo que ponerse en contacto con la Asociación de Emergencia de Científicos Alemanes en el Extranjero (Notgemeinschaft Deutscher Wissenschafter im Ausland) “que trabaja con el Consejo de Asistencia Académica de Londres (presidido por el conocido físico Lord Rutherford de Nelson)” (Lipschütz, 2 sept. 1936).

Ya sea por esta sugerencia o por otras agencias personales, Nehring logró salir de Alemania en 1937 hacia EEUU. Allí recibió ayuda de la Fundación Rockefeller, y en 1938 obtuvo un contrato en la Universidad de Marquette, en Milwaukee. En 1952 logró retomar el cargo que ocupaba en la Universidad de Würzburg, de la que llegó a ser rector entre 1953 y 1955 (Maas, 4 mayo 2018). La correspondencia aquí referida revela que desde 1936 Nehring estaba ansioso por salir de Alemania, y, a juzgar por las emociones que revelan sus cartas, muy desorientado y apesadumbrado por la situación. Su experiencia posterior refleja esta situación, ya que, como resume Utz Maas (4 mayo 2018), “Nehring fue uno de los innovadores de la década de 1920, para quien la expulsión se convirtió en un trauma que marcó su obra posterior”. Alfons Nehring falleció en 1967, en Würzburg. Algo de este doloroso proceso de exilio lo podemos constatar en esta rica correspondencia entre el lingüista alemán y el científico letón asentado en Chile, en busca de referencias y ayudas para huir del nazismo, eventualmente en dirección a Chile.

Algunos aspectos preliminares resultan importantes de destacar en este primer caso. Lipschütz se compromete en una agencia de carácter global a través de la cual imagina y propone a Nehring destinos como Madrid, Lisboa, Caracas, San José de Costa Rica, Buenos Aires, La Plata, la URSS. Al mismo tiempo, le advierte sobre el contexto poco auspicioso en América Latina, en la que ve universidades poco desarrolladas, disciplinas de rango académico y científico que son practicadas por aficionados, un recelo generalizado frente a profesionales extranjeros y una crónica falta de recursos. Además, Lipschütz procede con toda la energía y posibilidades de su red nacional y global, con lo que revela un profundo carácter humanitario, genuinamente interesado por el rumbo y la vida de los académicos alemanes atribulados en Alemania debido a las leyes racistas antisemitas. Se trata también de una comunicación entre académicos varones, imbuidos de su rol como científicos de prestigio y en el caso de Nehring, con un autoconcepto que no admitía otra posibilidad que la de ser el titular de una cátedra universitaria, algo que contrasta con la flexibilidad y apertura profesional y personal del siguiente caso registrado en el archivo personal de Lipschütz.

## La señorita Käte Pariser solicita ayuda: “Soy alemana, judía, 42 años y trabajo en citología y genética desde 1919”

Käte Pariser (1893-1953) es una figura bien conocida en la historia de los orígenes de la genética espanõla, debido a su permanencia como becaria en el Museo de Ciencias Naturales de Madrid (Gimber, Tovar, Moreno, 2010; Delgado Echeverría, 2007). Pariser obtuvo su doctorado en la Universidad de Berlín, en 1927, y era discípula de Richard Goldschmidt (1878-1958), director del Departamento de Genética y Biología Animal del Kaiser Wilhelm Institut für Biologie. Al culminar su doctorado, Pariser se integró al Institut für Vererbungsforschung de la Escuela Superior de Agricultura en Berlín-Dahlem y comenzó un programa de investigación sobre herencia e hibridación en distintas especies que le llevó a interesarse por un puesto en España para ampliar sus estudios a especies de la península ibérica. En 1933 tomó contacto con el doctor Antonio de Zulueta (1855-1971), un pionero de la genética española (Baratas, 1997; Galera, 2020), y con su apoyo y el de Goldschmidt postuló a una beca de la Asociación Universitaria Femenina de Madrid, que era una sección de la Federación Internacional de Mujeres Universitarias. Pariser emigró a Suiza sin saber el resultado de la beca, la que fue finalmente aprobada y permitió que iniciara sus investigaciones en Madrid en octubre de 1933, en el sótano del Museo de Ciencias Naturales. Ante el fin de su beca, y con plena conciencia de la situación en Alemania, comenzó la búsqueda de una nueva posición profesional a fines de 1935, como evidencia la carta que dirigió a Lipschütz el 17 de noviembre de ese año, por una recomendación del doctor Zulueta.

Pariser se presentó directamente como judía y dio a Lipschütz una breve referencia de su desarrollo científico, que culmina con una terrible constatación: “Ahora la oportunidad de trabajar en el extranjero se ha convertido en una necesidad para mí, ya que ya no puedo obtener un permiso de trabajo en ningún instituto en mi patria” (Pariser, 17 nov. 1935). La situación era tan urgente para ella que se presenta dispuesta incluso a rescindir su rol científico y señala que “también podría aceptar un trabajo que implique trabajar como secretaria” (Pariser, 17 nov. 1935) y que “le agradecería especialmente, estimado profesor, si fuera tan amable de indicarme si había alguna posibilidad de que me empleara en los institutos que usted conoce en Concepción o en algún otro lugar de Chile” (Pariser, 17 nov. 1935). Con humildad, Pariser, que estaba en la vanguardia de la genética, solicitaba a Lipschütz simplemente un trabajo técnico o de secretariado que le permitiera básicamente solventar sus gastos.

Lipschütz (8 ene. 1936) respondió a comienzos de 1936, ofreciéndole un cálido apoyo, pero mostrando un panorama poco auspicioso, indicando que “las condiciones no son favorables en América del Sur” y que, en Chile, particularmente “el ambiente para contratar extranjeros no es favorable. Falta también la seriedad necesaria, y, con ella, la posibilidad de apreciar correctamente el trabajo científico” (Lipschütz, 8 ene. 1936). A pesar de estas impresiones sombrías, Lipschütz le instó a que, presentándose con su recomendación, tomara contacto con Clemente Estable, director del Laboratorio de Ciencias en Montevideo, Uruguay, y con el doctor Afranio do Amaral, director del Instituto Butantan de São Paulo, Brasil, dándole las direcciones postales de ambos.

Pariser (2 feb. 1936) respondió a Lipschütz desde Berlín con una carta manuscrita en que reiteró que “tengo que buscar a toda costa un puesto de trabajo” y que estaba en gestiones para una beca en EEUU. Por su parte, Lipschütz acudió a las redes judías en Santiago de Chile y presentó el caso al médico judío, radicado en Chile, Oscar Koref, que, desde 1933, formaba parte de un comité de ayuda local para los judíos que lograban emigrar hasta Chile (Bohm, 1997). Lipschütz agregó un juicio rotundo sobre la situación política local en la Universidad de Concepción:

Perdóname por molestarte con tantas cosas nuevas. Desafortunadamente, aquí no se puede hacer nada en el acto, ya que en nuestra universidad ha estallado una verdadera cruzada contra los judíos y los izquierdistas. Hace unos días, un excelente profesor de matemáticas fue despedido sin motivo alguno; esta vez un chileno que trabajaba por contrato. Aquí solo hay una (buena) perspectiva para los elementos nazis (Lipschütz, 19 feb. 1936).

Luego de esta gestión, en el mes de marzo de 1936 Lipschütz respondió a Pariser con un tono pesimista respecto de la situación en Chile para la comunidad judía, especialmente en el ámbito universitario:

En Chile, se debe esperar animosidad hacia los extranjeros, especialmente judíos, principalmente en Concepción. Hay muchos alemanes aquí en Chile que tienen una mentalidad nazi y dada la gran influencia que estos círculos también han ganado en la universidad, las perspectivas para los judíos se ven perjudicadas (Lipschütz, 6 mar. 1936).

Si por una parte le advirtió claramente a Pariser que “solo deberías venir a Chile si no tienes otra opción” (Lipschütz, 6 mar. 1936), no dejó cabos sueltos en cuanto a brindarle una salida y le comunicó que:

Se le otorgará un permiso de ingreso a Chile a través de la mediación de la organización judía chilena. Al respecto, lo mejor es escribir al Sr. Oscar Koref, casilla 3528, Santiago, Chile. He mantenido correspondencia con él sobre su asunto y será de gran ayuda para usted (Lipschütz, 6 de mar. 1936).

Finalmente, igual que en el caso de Nehring, Lipschütz sugirió a Pariser que mirara hacia la URSS y se contactara con el profesor doctor H.I. Muller del Institut Genetique, Académie des Sciences de Moscú.

El último documento de la correspondencia entre Lipschütz y Pariser es la respuesta de la investigadora alemana, fechada en Madrid el 8 de abril de 1936. En esta misiva Pariser se muestra optimista respecto de una beca para trabajar en EEUU y menciona el respaldo de Zulueta, que la apoyaba con una beca para unos meses más en Madrid. Aunque se le estaban abriendo algunas perspectivas para EEUU, la científica volvió a señalar a Lipschütz que “la idea de ir a Sudamérica no es ni mucho menos un último recurso para mí, siempre pienso que con mis conocimientos de español sería útil allí” (Pariser, 8 abr. 1936). Ante el estallido de la guerra civil en España en julio de 1936, Pariser viajó a Tel Aviv, y luego regresó a Berlín tan solo para embarcar hacia Australia, país en el que fue docente universitaria desde fines de 1936 hasta su muerte, en 1953.

El caso de Käte Pariser revela aspectos de género destacables para el análisis, ya que su correspondencia con Lipschütz parece siempre mediada por la figura de su patrón científico en España, el doctor Zulueta; se dirige siempre a Lipschütz como a un maestro y su humildad, tanto como su necesidad, la llevan proponer su empleo en labores subalternas o técnicas. Así, podemos ver como Pariser adoptaba en su correspondencia una presentación y aspiraciones mediadas por su condición de mujer y científica y pensaba que al menos tan solo a partir de su conocimiento del idioma podía llegar a ser útil para sus hipotéticos empleadores sudamericanos.

## Consideraciones finales

Las gestiones de Lipschütz en torno a las solicitudes de Nehring y Pariser se ubican en un contexto de urgencia creciente por las persecuciones de parte del nazismo hacia los judíos y dentro de una gestión humanitaria internacional que apenas estaba poniendo sus bases (Löhr, 2014). En nuestro caso se trata de gestiones que surgen de agencias individuales y en las que se despliegan intimidad y emociones en el curso de gestiones y tentativas a veces con claras notas de desesperación. Se trata de unas relaciones relativamente horizontales, aunque con claras diferencias de género, y no unas relaciones totalmente asimétricas como las que se dieron entre refugiados solicitantes y las grandes instituciones de ayuda en EEUU. Además, se trata de gestiones en las que se encuentran dos éticas humanitarias; una de solidaridad judía y otra de solidaridad científica. Es notable como en torno al caso de Käte Pariser, Lipschütz acude prontamente a la solidaridad judía y la precave sobre el antisemitismo dominante en la sociedad chilena, mientras que con Alfons Nehring evita toda referencia al judaísmo, tal vez consciente de que se trata de un católico de madre judía. En ningún caso Lipschütz parece omitir iniciativas por esta diferencia, aunque las gestiona con diferencias estratégicas.

Sobre Lipschütz resulta necesario relevar su agencia personal en una red transnacional que involucraba contactos personales con científicos en Buenos Aires, La Plata, Montevideo, São Paulo, Caracas, Madrid, Berlín, Tartu, Moscú y, por supuesto, Santiago y Concepción, en Chile. Si bien esto nos habla de su excepcionalidad y relevancia en la endocrinología occidental, debemos recordar también que la universidad europea de inicios del siglo XX se basaba en gran medida en transferencias y conexiones que superaban las fronteras nacionales y que Lipschütz era un claro representante de ella, con profusas conexiones personales en el mundo de las ciencias biomédicas. Hay que anotar también que es una red épica en sus esfuerzos, pero con muy pocos logros, ya que de todas las gestiones realizadas por Lipschütz para disponer de un trabajo para científicos que emigraban de Alemania, los éxitos conocidos son muy pocos.

Aquí, cabe preguntarse si este fracaso en el logro de posiciones académicas o laborales es algo particular del tipo de gestiones personales de Lipschütz o bien si hay algunas condiciones del escenario local que no permitían mayores éxitos. Los casos para una posible comparación son pocos, ya que la migración de científicos judíos a Chile durante el nazismo es un tema poco estudiado. Así, podemos encontrar algunos casos ejemplares y virtuosos, mientras que otros muestran efectivamente unas condiciones estructurales resistentes a la incorporación de académicos y científicos judíos a universidad. Un caso excepcionalmente exitoso es el de Julio Hirschmann Recht, nacido en Bolivia de padres alemanes, que se formó como ingeniero en Universidad Técnica de Braunschweig y vivió en Alemania hasta 1932, año en que se trasladó a la URSS como académico de la Universidad de Leningrado (Escudero, 2014). En la monografía de Nelson Escudero dedicada a Hirschmann se comenta que “en 1937 la Universidad Técnica Federico Santa María, desde Chile, le reclutó como parte del plan de contratación docente que buscaba profesores alemanes, aunque dada su condición de judío no se deben descartar otros factores” (Escudero, 2014, p.17); de ello no resulta claro el contexto de su contratación y las fuentes citadas remiten a comunicaciones orales. En todo caso Hirschmann llegó a ser decano de la Facultad de Mecánica en 1944, vicerrector de la Universidad en 1965 y un pionero de la energía solar a nivel global, por lo que se trata de un caso excepcionalmente exitoso y sin mayores trabas a su rápida integración. Otros casos, como el de Heinrich Finkelstein (1865-1942) nos presentan un panorama distinto y lleno de precariedad. Finkelstein fue uno de los pioneros de la pediatría en Alemania, una disciplina especialmente afín a los médicos judíos alemanes en las primeras décadas del siglo XX (Saenger, 2006). Se trata de un médico de renombre internacional, promotor de un cuidado integral de la infancia y creador junto a Ludwig Ferdinand Meyer de la primera leche modificada artificialmente a partir de la proteína de la leche de vaca que logró a salvar a miles de niños en Alemania y el mundo. A pesar de su renombre internacional, las leyes nazis le quitaron la licencia para ejercer la medicina en 1936. Las batidas antisemitas de 1938 le llevaron a buscar refugio en forma urgente, momento en que un discípulo chileno, el médico Arturo Scroggie, le ofrece la posibilidad de venir a Chile. La situación de Finkelstein en Chile resulta trágica. Sabemos que en principio le fue otorgada una pensión honoraria, la que, con un cambio en el gobierno, le fue retirada. Para salvar la situación, colegas de la Universidad de Chile le crearon un contrato como portero del Hospital, a través del cual seguían acudiendo a él en casos difíciles hasta su fallecimiento, en 1942. El caso del exilio de Finkesltein es bien conocido pero muy poco estudiado en sus detalles; de todas maneras llama la atención que una autoridad mundial en pediatría, a falta de otras palabras, fuera humillado en forma tan directa en Chile. Y esto nos lleva justamente a un problema para el exilio científico judío en Chile por aquellos años: el antisemitismo. Al describir a los posibles emigrantes un panorama sobre Chile, Lipschütz ofreció algunas imágenes pesimistas sobre la inmadurez y falta de seriedad profesional en las disciplinas científicas en la sociedad y en la universidad chilena. Además, reportó un antisemitismo generalizado y con mucha influencia tanto en la sociedad chilena como en la Universidad de Concepción. Sabemos con certeza que el nazismo local era muy activo (Farías, 2000) y que el sentimiento anticomunista, antisemita y de simpatía con el nazismo triunfaba en la comunidad alemana residente y en algunos sectores profesionales y confesionales chilenos (Farías, 2000; Sánchez Delgado, 2014). En particular, importantes sectores de la medicina chilena cultivaron una creciente simpatía por el nazismo y se integraron a las actividades científicas como fue el caso de la Academia Médica Germano Iberoamericana, institución especialmente creada para fortalecer los vínculos del nazismo con los médicos iberoamericanos. En el ámbito privado, las declaraciones pronazis de algunos médicos chilenos podían llegar a ser admirativas e incluso vociferantes, como las de Ernesto Prado Tagle que lamentaba que en Chile “no contamos con un Hitler a quien admirar”, o las de Juvenal Barrientos, que opinaba que “me creo más nacista que el mismo Hitler, de quien creo que con solo exterminar a los judíos de Alemania habrá cumplido su obra en demasía” (citados por Sánchez Delgado, 2018, p.371). Todo lo anterior nos ofrece un panorama indiscutiblemente pronazi y antisemita en buena parte de la medicina chilena, lo que se transformaba en un factor a veces insuperable para operar en favor de los médicos y científicos judíos exiliados. Por otra parte, también es cierto que amplios sectores sociales permanecieron ajenos al antisemitismo y hay trabajos que llegan a señalar una buena acogida para los migrantes judíos en los ambientes de clase media chilena de la época (Goldschmidt, 2016).

Las iniciativas de Alfons Nehring y de Käte Pariser tendientes a lograr una posición profesional en Chile no se concretaron y a pesar del optimismo de Pariser en torno a que Sudamérica no era tan solo un último recurso, los destinos de ambos se unieron a otros espacios nacionales. Nehring emigró a EEUU y Pariser a Australia. Estas posibilidades no estaban ni siquiera insinuadas en la correspondencia con Lipschütz, pero si la sugerencia a Nehring de contactar con la Notgemeinschaft y la rápida gestión de Lipschütz por integrar el caso de Pariser a un entorno de solidaridad judía. Alfons Nehring volvió a Alemania y llegó a ser rector de la Universidad de Wurzburg, mientras que Käte Pariser logró posiciones académicas en Camberra, primero, y luego en Sidney. En todo caso, Nehring nunca superó el trauma del exilio, y Pariser se ligó a la docencia y perdió totalmente el lugar de relevancia y vanguardia científica que había logrado tanto en Berlín como en Madrid gracias a sus investigaciones. A través de estas cartas y trayectorias vemos las consecuencias dolorosas de la política antisemita en la ciencia y en la universidad alemanas, en dos personas cuyo rumbo vital resultó alterado por una violencia racista estructural y social de su época.

Este trabajo también nos permite acercarnos a una faceta desconocida de Alejandro Lipschütz y nos lleva a considerarlo como uno de los centros de gravedad sudamericanos de una red de apoyo a los científicos judíos que huían del nazismo. Las cartas que circularon entre Lipschütz, Nehring y Pariser nos hablan de luchas, esperanzas, miedos, urgencias y tribulaciones del pequeño mundo académico, puesto en una dimensión épica de vida o muerte, que no suele ser la propia de profesores y de los científicos y científicas, sumidos en tareas en sus laboratorios. El nazismo empujó aquellas trayectorias hacia un periodo trágico y lleno de angustias que resultaron lamentablemente justificadas. Lipschütz también terminaría siendo afectado directamente por el genocidio perpetrado contra los judíos en Europa, ya que en correspondencia privada del período de posguerra señaló el asesinato de casi la totalidad de sus parientes que residían en Riga por causa del antisemitismo genocida del nazismo.

La historiografía anglosajona y europea conoce muy bien las vidas de los grandes científicos judíos exiliados de Alemania desde 1933 en adelante y también la historia de las grandes fundaciones filantrópicas que ayudaron a derribar las barreras de entrada a la universidad inglesa y norteamericana (Lamberti, 2006). Nuestro trabajo presenta una historia de agencias personales y localizadas en el contexto de un país sudamericano y periférico, sin recursos, sin fundaciones y con una gran tensión política interna; país que la señorita Käte Pariser y el profesor Alfons Nehring veían como un posible lugar en el que vivir libremente y desarrollar las actividades para las que se habían preparado durante toda su vida y que les daban tanto el sustento material como el sentido vital; país en el que Lipschütz seguía siendo un ciudadano del ancho mundo de la ciencia y la cultura y estaba dispuesto a proteger y ayudar a los suyos en la ciencia y en la comunidad, así como a luchar contra el nazismo, desde su máquina de escribir.

En el contexto de un inagotable interés por el nazismo, las redes de solidaridad latinoamericana están siendo parte de un renovado interés historiográfico preocupado por los conocimientos migrantes que originaron estos viajes transnacionales. Nuestro interés ha sido presentar, analizar y contextualizar un material epistolar inédito y colaborar al estudio de las redes latinoamericanas del exilio científico judío alemán.

En gran medida esta investigación está motivada por la convivencia cotidiana con las carpetas del archivo personal de Alejandro Lipschütz. Las cartas y documentos utilizados nos han puesto en contacto con las angustias de científicos que ansiaban llegar a un lugar seguro en que continuar con sus vidas y, aun sin saber a ciencia cierta lo que sucedería, terminar salvándola. Son cartas manuscritas, mecanografiadas originales o en copias en calco en hojas casi transparentes que están a punto de disolverse en nuestras manos. Por ello, junto a las más nobles y sofisticadas tareas del historiador o historiadora, nos interesa también y de manera legítima, documentar, transcribir en parte y presentar estos documentos a la experiencia del lector, al mismo tiempo que narrar y contextualizar esta historia. Parafraseando a Kracauer, nos hemos encontrado con un material que nos reclamaba situarnos en las últimas cosas antes de las últimas; en otras palabras, abandonar el ego académico e intentar dar protagonismo a la experiencia anidada en esas cartas.

Por otra parte, en el interés general por el nazismo y sus fenómenos de exilio, persecución y violencia racista puede darse, pensamos, una pregunta por la actualidad, que un autor como Carl Amery (2002) resumió en la inquietud de reflexionar si el fenómeno del nazismo se encuentra clausurado en la experiencia histórica del siglo XX o pudiera llegar a ser un fenómeno precursor del siglo XXI.

## NOTAS

1 En esta y otras citas de otros idiomas, se ha facilitado una traducción libre.

2 Se menciona también el esfuerzo del profesor Laqueur por ellos. Resulta necesario mencionar que Ernst Laqueur (1880-1947) fue uno de los primeros científicos que logró producir insulina en grandes cantidades y también logró aislar el estrógeno y la testosterona. Cuando los nazis ocuparon Holanda, perdió su cátedra por su origen judía e intentó salir de Europa sin éxito. Él y sus dos hijas fueron liberados por tropas británicas y soviéticas desde Bergen-Belsen en 1945.

3 Ernst Lewy (1881-1966) fue profesor titular de la Universidad de Berlín desde 1925, exonerado por las leyes de 1933 y reintegrado por un breve periodo hasta su expulsión definitiva en 1935. Gracias a la ayuda del egiptólogo británico Alan Gardiner emigró a Irlanda en 1937. Por su parte, Max Vasmer mantuvo su cátedra en Berlín junto a una intensa actividad como conferencista en EEUU. En 1944, su casa en Berlín fue bombardeada y perdió gran parte de su biblioteca; sin embargo, logró publicar una obra magna sobre etimología eslava en tres volúmenes entre 1950 y 1958, obra que se considera autoridad hasta la fecha.
